# Indents

**Published:** 1912-09-15

**Authors:** 


### INDENTS.

☞Brief Articles of Technical or Scientific Value will receive notice and comment. Contributions invited.

*Quackery Abroad.* Quackery flourishes the world over. European countries, where on account of strong paternal governments many things are managed well, are not free from this pest. Quacks thrive in England, Germany and other countries of Europe, but benighted Russia seems to keep them in leash better than most other countries. As far back as 1788, laws were provided in Russia protecting the people from all forms of irregular practice, and such laws have continued to hold a restraining hand on quackery down to the present time. By the application of a well-known modern political method, however, a "joker," which affords a loophole for a certain amount of irregular practice, has been inserted into some of the laws regulating the practice of medicine. Still, the number of quacks in Russia who do business on a large scale is small.

In Germany during the early part of the nineteenth century, laws against quackery were stringent and effective, but curiously enough, at the instance of the medical society of Berlin in 1869, the regulations against irregular practice were much relaxed and the result was a tremendous development of quackery in Germany. Berthenson says that in 1869 the number of quacks in Berlin was twenty-eight. In twenty-four years the number was over a thousand and the whole number practicing in Prussia was over five thousand. In certain districts the number of irregulars outnumbered the qualified practitioners, two to one. In 1906 it was estimated that the number of quacks in Prussia was 10,000 and the scope of their practice had become unlimited. The unqualified practitioners are regularly organized and have schools and institutions providing a four months' course for quacks; there are over 800 societies for study in "nature healing" with a total membership of 112,000. There are over fifty periodicals with millions of circulation. The quacks come largely from the uneducated class, and it is said that over 58 per cent. of the female irregulars have been domestic servants. This condition has led to efforts to amend the laws in such a way as to limit the practice of these people to the minor ills. This has led to strongly organized opposition, similar to the League for Medical Freedom in our country, and, as in the case of our own Congress, the opposition has found support in the Reichstag. This feeling was shown by the chilly reception which the first reading of the proposed legislation received in that body, the same reason being ascribed as here, namely, that the measure would create a "medical trust."

The forces of graft and unrighteousness are peculiar to no country or clime, and they have their champions in the high places and the low. Until the people themselves are better educated concerning the danger and inequity of quackery, they must be protected from the forces that prey. The popular understanding of these matters is becoming better every day, and, aided by proper laws, the time will come, perhaps, when quackery will be unprofitable.—*Journal A. M. A.*, June 8, 1912.

*Women in Dentistry.* Women thinking of entering the dental profession will be interested to learn that there has recently been instituted a five year course for women students for the study of dentistry and in preparation for the License in Dental Surgery of the Royal College of Surgeons of England, at the London (Royal Free Hospital) School of Medicine for Women and the National Dental Hospital, Great Portland Street, W. The council of the London School of Medicine for Women will award annually (until further notice) an "Agnes Guthrie" Bursary, of the value of 60 Pounds, to a student fulfilling the required conditions who enter for the full dental course. The first award will be made at the end of September, 1912, for the course beginning in October, 1912. Candidates for the bursary are required to send in applications on or before Sept. 15th, 1912, to the Secretary and Warden, 8 Hunter Street, Brunswick Square, W.C., from whom all particulars can be obtained. The combined fees at the London (Royal Free Hospital) School of Medicine for Women and the National Dental Hospital are 180 Pounds if paid in one sum on beginning the course, or 191 Pounds if paid in three annual installments.—*Dental Record.*

*Incorporating Metal in Wax Model.* In casting work where a portion of metal is to be incorporated in the wax model there is always difficulty in making the metal stick to the inlay wax. This may be readily overcome by coating the metal on the side or sides that are to come in contact with the inlay wax, with one of the good "sticky waxes." This makes the metal readily incorporate with the inlay wax and causes no trouble whatever in the subsequent operation of casting—Dr. Arthur G. Smith, in *Review.*

